# Regulatory Mechanisms Underlying Stem Strength and Toughness in Dicotyledonous Plants: Implications for Soybean Breeding

**DOI:** 10.3390/cimb48020189

**Published:** 2026-02-07

**Authors:** Ye Zhang, Elshan Musazade, Javaid Akhter Bhat, Songling Xie, Yaohua Zhang, Weitao Xu, Xianzhong Feng, Suxin Yang

**Affiliations:** 1 Key Laboratory of Soybean Molecular Design Breeding, Northeast Institute of Geography and Agroecology, Chinese Academy of Sciences, Changchun 130102, China; zhangye222@mails.ucas.ac.cn (Y.Z.); elshan.musazade1@gmail.com (E.M.); javid.akhter69@gmail.com (J.A.B.); xiesongling24@mails.ucas.ac.cn (S.X.); zhangyaohua@iga.ac.cn (Y.Z.); xuweitao@iga.ac.cn (W.X.); fengxianzhong@iga.ac.cn (X.F.); 2 College of Advanced Agricultural Sciences, University of Chinese Academy of Sciences, Beijing 101408, China

**Keywords:** soybean, stem, toughness and strength, vascular bundle, secondary cell wall (SCW), cellulose, hemicellulose, lignin

## Abstract

Soybean (*Glycine max*) is a globally important crop valued for its high seed oil and protein content. However, lodging remains a major abiotic constraint that causes substantial yield losses. Lodging resistance is primarily determined by stem strength and toughness, which are governed by stem anatomical organization, vascular tissue development, and the composition and architecture of secondary cell walls (SCWs). This review synthesizes current knowledge on anatomical, structural, and genetic factors that are implicated in stem mechanical performance in dicotyledonous plants, with particular emphasis on vascular cambium activity, xylem and phloem differentiation, and the biosynthesis of major SCW components, including cellulose, hemicellulose, and lignin. These processes collectively determine stem rigidity, flexibility, and resistance to mechanical stress. By integrating insights from model species, especially *Arabidopsis thaliana*, and non-soybean dicots, this review highlights conserved regulatory pathways controlling stem development and SCW formation that are directly relevant to soybean improvement. The synthesis provides a translational framework for understanding how conserved anatomical and genetic mechanisms can be leveraged to enhance soybean stem strength, toughness, and lodging resistance. Overall, this review provides a conceptual foundation for future functional studies and breeding strategies to improve soybean yield stability and adaptability across diverse agronomic conditions.

## 1. Introduction

Soybean (*Glycine max*) is one of the world’s most important crops, serving as a major global source of vegetable oils and plant-based proteins and providing essential raw materials for food processing and animal feed industries [1]. With continued global population growth and increasing demand for meat and dairy products, the sustained improvement of soybean productivity and yield stability has become an urgent agricultural priority [2]. Although global soybean production has increased substantially over recent decades, further gains are required to meet future food and feed demands, particularly under intensive cultivation systems [1].

In cereal crops such as wheat and rice, yield improvements have been achieved through the development of semi-dwarf varieties that enhance lodging resistance and enable higher planting densities [3]. However, soybean exhibits a distinct dicotyledonous growth habit, characterized by the formation of leaves, inflorescences, and pods at each node along the stem [4]. Consequently, soybean yield is closely linked to plant height, internode number, and node development [5]. Unlike cereals, excessive reduction in soybean plant height can negatively affect node formation and reproductive potential, presenting a fundamental challenge for increasing planting density without compromising yield components.

High planting density in soybeans often leads to reduced stem strength, exaggerated internode elongation, and a markedly increased risk of lodging [6]. Lodging is a major constraint on soybean yield and typically manifests as either stem bending or basal stem fracture [6,7]. Stem bending primarily results from insufficient structural strength, whereas basal fracture reflects inadequate mechanical toughness [6,7]. Together, these observations highlight stem strength and toughness as critical traits influencing lodging sensitivity and yield stability in densely planted soybeans.

The stem plays a central role in plant growth and performance by providing mechanical support, facilitating long-distance transport, enabling storage, and protecting vascular tissues [8]. As a typical dicotyledonous plant, soybean undergoes secondary growth, resulting in a complex stem structure composed of epidermal, cortical, vascular, and pith tissues [9]. Despite the importance of stem mechanical properties for agronomic performance, the genetic and regulatory mechanisms controlling stem development and mechanical integrity in soybean remain poorly understood.

In contrast, extensive studies in *Arabidopsis thaliana* and other dicotyledonous model plants have elucidated key anatomical, cellular, and molecular mechanisms governing vascular development, secondary cell wall (SCW) formation, and stem mechanical properties [10]. This review systematically examines the structural components and regulatory networks underlying stem strength and toughness in dicotyledonous plants. By integrating conserved pathways and genetic regulators identified in model systems, we propose a translational framework to inform future functional studies and breeding strategies to enhance soybean stem strength, toughness, and lodging resistance. Overall, this review provides a conceptual foundation for improving soybean yield stability and adaptability under diverse agronomic conditions.

## 2. Key Tissues and Structural Components Underlying Stem Strength and Toughness in Dicotyledonous Plants

### 2.1. Tissue-Level Anatomical Determinants of Stem Mechanical Strength

**Figure 1:**
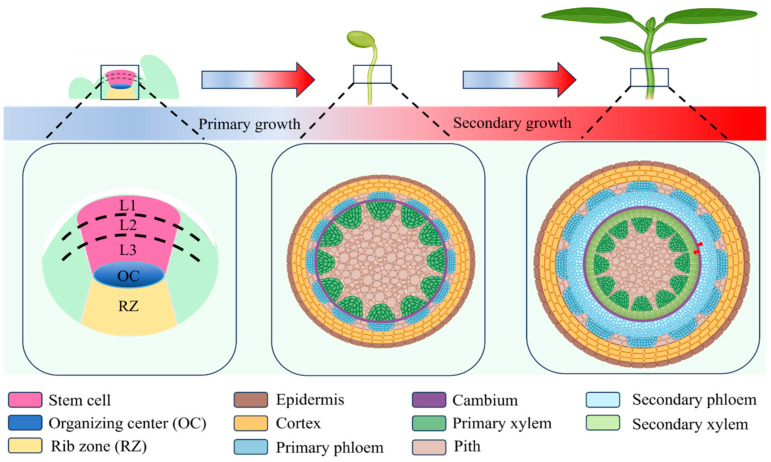
Developmental process of dicotyledonous plant stems. In many dicotyledonous plants, the shoot apical meristem (SAM) exhibits distinct cytological organization, typically comprising three well-defined layers: L1, L2, and L3. Through primary growth, the SAM establishes the basic structure of the stem, while secondary growth subsequently enables the stem to undergo thickening. [Created in BioRender. Ye, Z. (2026) https://BioRender.com/uolji88 (accessed on 22 January 2026)].

The mechanical properties of dicotyledonous stems are closely related to their anatomical structure, which is evident at multiple levels, including the vascular bundle system, cell wall composition, and cellular morphology. A typical dicot stem consists of the epidermis, cortex, vascular bundles, and pith (Figure 1). Acting as the structural framework of the stem, vascular bundles play a dual role in long-distance transport and mechanical reinforcement. The primary vascular bundles are located in the inner region of the cortex that originates from the procambium within the L3 layer of the shoot apical meristem (Figure 1) [11,12,13,14]. These vascular bundles comprise primary phloem, primary xylem, and vascular cambium [11]. The primary phloem is composed of sieve tubes, companion cells, phloem parenchyma, and phloem fibers. The function of primary phloem is the long-distance transport of organic compounds produced especially during photosynthesis to the other parts of plants [12].

In contrast, the primary xylem consists of vessels, tracheids, xylem parenchyma, and xylem fibers and functions primarily in the long-distance transport of water and inorganic nutrients from the root system to the aerial parts of the plant [12]. Beyond its transport role, the xylem provides critical mechanical support, as vessels and xylem fibers possess thick SCWs enriched in cellulose, hemicellulose, and lignin, which collectively confer rigidity and enable sustained upward growth [13].

The vascular cambium is located between the primary phloem and the primary xylem. It originates from incompletely differentiated procambial cells [11]. The vascular cambium produces secondary xylem toward the interior, which is composed of vessels, tracheids, wood fibers, and xylem parenchyma cells (Figure 1) [14,15]. The vascular cambium produces secondary phloem toward the exterior, which consists of sieve tubes, companion cells, phloem fibers, and phloem parenchyma cells (Figure 1) [14,15]. The aforementioned division and differentiation processes in the vascular cambium play a critical role in mediating the radial expansion of the stem [16]. Stem diameter is positively correlated with bending strength and lodging resistance [5].

Beyond overall stem morphology, the mechanical contribution of vascular tissues is primarily determined by SCW formation in xylem and phloem fibers. The chemical composition, thickness, and structural integrity of these secondary walls are critical determinants of stem stiffness, toughness, and resistance to bending forces [17,18]. Moreover, the concentric organization of the pericycle-phloem complex exhibits a high degree of structural similarity across stems, hypocotyls, and roots, and is regulated by conserved genetic programs governing vascular development and tissue patterning. Collectively, these shared anatomical and regulatory features underpin the concept of vascular tissues in roots, hypocotyls, and stems being functionally unified as “wood”, reflecting their common role in mechanical support across plant organs [19,20].

Collectively, these observations demonstrate that stem mechanical strength in dicotyledonous plants arises not only from overall stem diameter but also from the coordinated development of xylem and phloem fibers, SCW deposition, and conserved vascular architecture. The vascular bundle system, therefore, represents a fundamental anatomical determinant of stem strength, providing the structural stability and resilience required for upright growth and successful reproduction.

### 2.2. Cell Wall Structural Components Governing Stem Strength and Toughness

**Figure 2:**
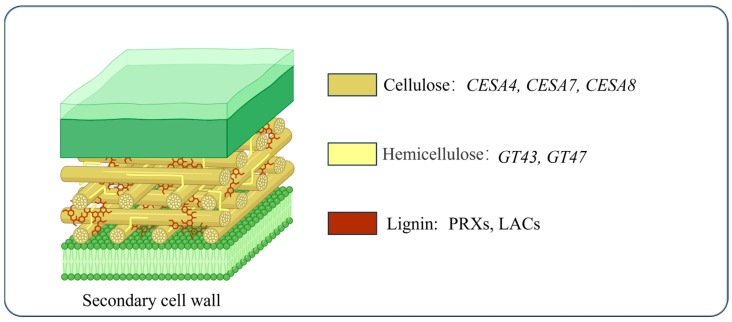
Structural composition of the SCW. The key genes involved in cellulose biosynthesis are cellulose synthase subunits *CESA4*, *CESA7*, and *CESA8*. Multiple Golgi-localized glycosyltransferase families, including *GT43* and *GT47*, are involved in the biosynthesis of the hemicellulose backbone and the addition of side chains. Key enzymes involved in lignin biosynthesis include laccases (LACs) and peroxidases (PRXs). [Created in BioRender. Ye, Z. (2026) https://BioRender.com/fzk0hsu (accessed on 22 January 2026)].

The development of SCWs is a central determinant of stem mechanical strength and toughness in dicotyledonous plants. SCWs are primarily composed of three major structural components, cellulose, hemicellulose, and lignin, whose relative abundance, molecular organization, and spatial arrangement collectively define the mechanical behavior of plant stems (Figure 2) [21]. Together, these polymers confer structural rigidity, resistance to deformation, and the capacity to withstand mechanical stresses, while also supporting efficient water and nutrient transport within vascular tissues [22,23,24].

Cellulose, the most abundant structural polysaccharide, forms a highly ordered microfibrillar network that provides the principal tensile strength of the cell wall. Disruption of or reduction in cellulose synthesis often results in xylem collapse and pronounced weakening of stem mechanical integrity, underscoring its essential structural role [25]. Hemicellulose, synthesized in the Golgi apparatus as branched polysaccharides such as xylan and glucomannan, is transported via vesicles to the plasma membrane and incorporated into the SCW, where it crosslinks cellulose microfibrils through hydrogen bonding, thereby enhancing wall cohesion and flexibility [26].

Lignin synthesis constitutes the final stage of SCW formation and plays a decisive role in determining wall stiffness, hydrophobicity, and resistance to compressive forces. Lignin is deposited through the oxidative polymerization of three principal monolignols, sinapyl alcohol (S unit), coniferyl alcohol (G unit), and *p*-coumaryl alcohol (H unit), catalyzed primarily by peroxidases (PODs) and laccases (LACs) within the SCW, thereby filling interstitial spaces between cellulose-hemicellulose networks and reinforcing the wall matrix [27,28,29]. Numerous studies have demonstrated that both lignin content and monomer composition are tightly correlated with cell wall hardness and mechanical strength [30]. In crops, increased lignin accumulation in stems significantly enhances mechanical support and lodging resistance [31].

Collectively, cellulose and lignin are the dominant contributors to stem mechanical strength, while hemicellulose modulates wall architecture and flexibility. Reductions in cellulose or lignin levels increase stem brittleness, rendering plants more susceptible to bending, breakage, and lodging [32]. Consequently, lodging severity and incidence are closely linked to alterations in stem mechanical properties, and strengthening stem tissues has been shown to reduce yield and quality losses under adverse environmental conditions [33,34].

At the anatomical level, enhanced stem mechanical performance is associated with thicker cell walls, increased vascular bundle number, well-developed parenchyma tissues, and elevated lignin deposition within SCWs. These structural and compositional traits have been consistently observed in crops and model species exhibiting superior stem strength and lodging tolerance [35,36]. Overall, coordinated regulation of SCW biosynthesis and architecture is fundamental to the development of stem strength and toughness in dicotyledonous plants.

## 3. Genetic Regulators of Stem Strength and Toughness in Dicotyledonous Plants

### 3.1. Genetic Regulation of Vascular Cambium Development

**Figure 3:**
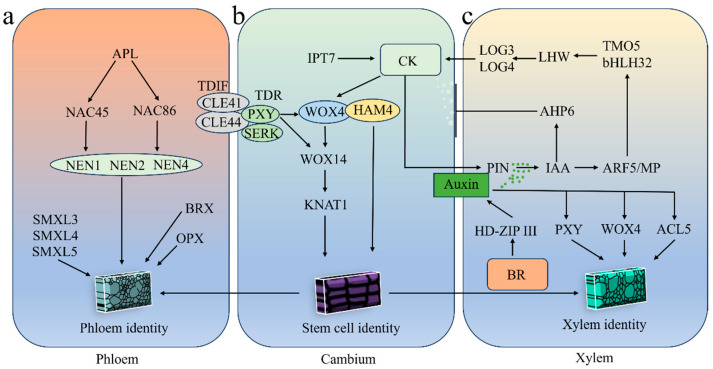
Regulatory network of vascular bundle development (**a**) Phloem development gene regulatory network. (**b**) Cambium development gene regulatory network. (**c**) Regulatory network of xylem development genes.

Vascular bundle development is a complex process involving multiple sequential stages, including procambial differentiation, formation of primary vascular bundles, and initiation of secondary growth [37]. At the molecular level, this process is tightly regulated by a diverse array of signaling molecules and transcription factors (TFs). The tracheary element differentiation inhibitory factor (TDIF)-TDR-WOX signaling pathway is essential for maintaining vascular cambium activity in the model plant *Arabidopsis*, and is widely conserved across the vascular plants (Figure 3b) [13,38,39]. TDIF is a phloem-derived dodecapeptide ligand, derived from CLAVATA3/ESR RELATED 41 (CLE41) and CLE44 [37].

Multiple members of the phloem intercalated with xylem (PXY) and somatic embryogenesis receptor kinase (SERK) kinase families function as receptors for TDIF, specifically as TDIF receptor (TDR). Activation of the TDIF-TDR complex promotes the expression of downstream TFs, notably *WOX4* and *WOX14*, which are key regulators of cambial cell proliferation and vascular bundle thickening in dicot stems (Figure 3b) [40]. Genetic analyses in *Arabidopsis* have demonstrated that co-activation of *WOX4* and *WOX14* leads to enhanced cambial activity and increased vascular tissue production, whereas loss-of-function mutants exhibit reduced cambial proliferation [40,41]. According to previous studies, the double mutant of *wox4 knat1* exhibits a complete loss of cambium activity at specific stem positions [41], indicating that *KNAT1* and *WOX4* are key determinants of cambium establishment. These findings highlight the importance of coordinated transcriptional control in regulating vascular development.

The hairy meristem gene family provides another regulatory layer in dicot stem development [3]. In *Arabidopsis*, higher-order *ham* mutants exhibit pronounced vascular abnormalities that resemble but exceed those observed in *wox4* mutants, indicating that HAM proteins regulate cambial activity through both WOX4-dependent and independent mechanisms [42]. This suggests that cambial regulation involves multiple partially overlapping transcriptional modules.

Phytohormone signaling is closely integrated with transcriptional regulation of the vascular cambium. Auxin signaling modulates cambial cell sensitivity and patterning, in part by regulating Auxin/indole-3-acetic acid (AUX/IAA) gene expression [43]. Cytokinins (CKs) represent another class of phytohormones that play pivotal roles in cambial cell differentiation, maintenance of proliferative activity, and cambium establishment (Figure 3b) [44,45]. Overexpression of the CK biosynthesis gene *IPT7* (isopentenyl transferase 7) accelerates cambium cell division [46].

Collectively, these studies in dicotyledonous model systems establish a conserved regulatory framework for vascular cambium development, involving peptide signaling, TF networks, and hormone-mediated control. This framework provides a foundational reference for understanding stem development across dicot species and for guiding translational research in crop improvement.

### 3.2. Genetic Regulation of Phloem Development

The physiological functions and structural integrity of the phloem indirectly influence the mechanical properties of the stem. The phloem transports photosynthetically derived compounds, including carbohydrates and hormones, from source tissues in leaves to growing regions of the stem, thereby providing the essential materials and energy required for vascular bundle development and SCW biosynthesis. Impairment of phloem transport function leads to a deficiency in precursors required for lignin and cellulose synthesis, which not only compromises thickening of phloem fiber cell walls but also inhibits xylem development, ultimately reducing the overall strength and toughness of the stem. The current research finds that the proteins encoded by the *BREVIS* (*BRX*) and *OCTOPUS* (*OPS*) genes are both localized to the apical region of developing protophloem cells and are essential for maintaining phloem continuity [47,48]. Altered phloem development (APL) is an MYB TF expressed in both sieve tube elements and companion cells, and plays a critical role in the proper differentiation of phloem tissues [49]. In addition, *APL* regulates sieve element enucleation by directly modulating *NAC45* and *NAC86* transcription (Figure 3a) [50]. *NAC45* and *NAC86* coordinate processes, such as enucleation and cytosol degradation, by modulating the expression of a set of exonuclease genes, including *NEN1*, *NEN2*, and *NEN4* (Figure 3a) [50]. Furthermore, *SMAX1-LIKE 3* (*SMXL3*), *SMXL4*, and *SMXL5* have been demonstrated to function as crucial, redundant regulators of phloem formation (Figure 3a) [51]. *smxl3 smxl4 smxl5* triple mutants are fully devoid of phloem tissue [52].

Collectively, studies in dicotyledonous model systems identify BRX, OPS, APL, NAC, NEN, and SMXL gene families as core components of the regulatory network governing phloem differentiation and maintenance. These conserved regulators provide a mechanistic framework for understanding phloem development and its contribution to stem structural integrity across dicot species.

### 3.3. Genetic Regulation of Xylem Development

Xylem tissue constitutes the principal load-bearing component of the vascular system in dicotyledonous stems. Its developmental status, including tissue organization, cellular architecture, and SCW properties, directly determines the mechanical support capacity of the stem. In addition, xylem development operates in close coordination with phloem differentiation, enabling an integrated balance between rigidity and flexibility that is essential for maintaining stem stability and resistance to mechanical stress [53].

The regulatory mechanisms governing xylem development in *A. thaliana* are well characterized. During xylem development in *Arabidopsis*, the auxin and CK signaling pathways coordinately regulate xylem pattern formation and cell differentiation through both antagonistic and synergistic interactions (Figure 3c). Progenitor cells in the primary xylem exhibit increased auxin signaling, which activates the expression of CK biosynthesis genes *LONELY GUY3* (*LOG3*) and *LOG4* via a TF-mediated cascade (Figure 3c). The targets include auxin response factor 5/monopteros (ARF5/MP) and the basic helix-loop-helix (bHLH) TFs target of monopteros 5 (TMO5) and lonesome highway (LHW) (Figure 3c) [54,55,56]. In contrast, the gene encoding the CK signaling inhibitor Arabidopsis histidine phosphotransfer protein 6 (AHP6) is upregulated (Figure 3c) [54,55,56].

Consequently, CKs accumulate in the procambial cells adjacent to the protoxylem precursor cells, stimulating cell proliferation and facilitating PIN-FORMED (*PIN*)-mediated auxin transport to the developing protoxylem tissues (Figure 3c) [54,55,56]. The auxin gradient within the xylem promotes the expression of *PXY* and *WOX4* in xylem progenitor cells (Figure 3c). The Class III Homeodomain Leucine Zipper (HD-ZIP III) TF family is predominantly expressed in cambium and xylem precursor cells, where it promotes xylem differentiation (Figure 3c) [57]. Phytohormones, such as auxin and brassinosteroids (BRs), also contribute to xylem differentiation by regulating the expression of HD-ZIP III TFs [58].

In addition to classical phytohormones, thermospermine is a novel plant growth regulator that represents an additional class of molecules that modulate xylem differentiation (Figure 3c) [59]. The gene encoding thermospermine synthase, *ACAULIS 5* (*ACL5*), is predominantly expressed in the xylem precursor cells. Furthermore, *acl5* mutant plants exhibit a dwarf phenotype, whereas exogenous application of thermospermine restores the wild-type phenotype (Figure 3c) [60,61]. Auxin also induces *ACL5* expression, whereas elevated thermospermine concentrations inhibit auxin-mediated xylem differentiation (Figure 3c) [58,62]. This suggests that plants regulate proper xylem differentiation via a negative feedback mechanism involving auxin and thermospermine.

Previous studies have demonstrated that NAC-domain protein 6 (*VND6*) and *VND7* serve as key regulators of xylem vessel differentiation [63]. Overexpression of *VND6* and *VND7* in *Arabidopsis* and woody dicot species such as poplar induces ectopic differentiation of xylem vessel elements in multiple tissues, underscoring their conserved roles in secondary wall formation and programmed cell death during xylem development [63,64,65].

Collectively, these studies establish a conserved regulatory network governing xylem development in dicotyledonous plants, integrating peptide signaling, TF cascades, phytohormone interactions, and metabolic feedback mechanisms. This framework provides a comprehensive reference for understanding the molecular basis of xylem differentiation and its contribution to stem mechanical properties across dicot species.

### 3.4. Genetic Regulation of Secondary Cell Wall Development

**Figure 4:**
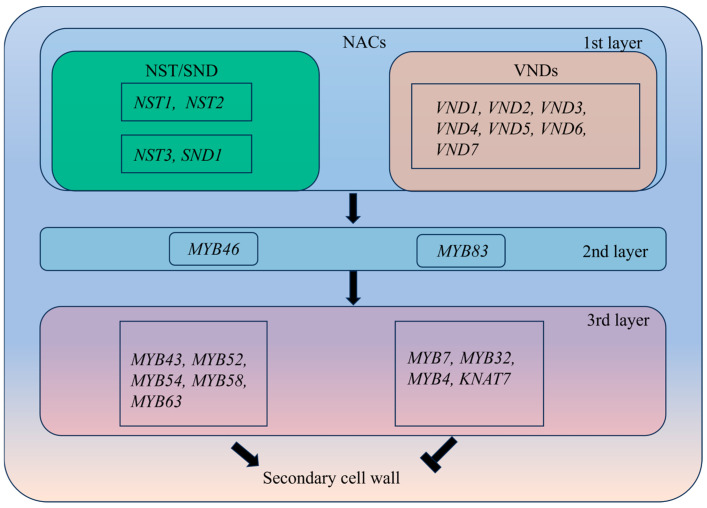
Regulatory network of SCW development. The key regulatory genes of the NAC family in the primary transcriptional network include NST and SND. The second tier of the transcriptional regulatory network governing SCW biosynthesis is primarily regulated by *MYB46* and *MYB83*. The third tier of the transcriptional regulatory network is primarily regulated by *MYB43*, *MYB52*, *MYB54*, *MYB58*, *MYB63*, *MYB7*, *MYB32*, *MYB4* and *KNAT7*. “
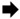
” indicates transcriptional activation; “
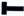
” indicates transcriptional inhibition.

The SCW represents the principal structural layer formed during the maturation of specialized stem cells, including xylem vessels, xylem fibers, and phloem fibers. Its developmental status, encompassing the timing of deposition, wall thickness, compositional ratios, and spatial organization, constitutes a central intrinsic determinant of stem strength and toughness in dicotyledonous plants. In contrast to the primary cell wall’s “flexible support” function, the SCW provides fundamental mechanical rigidity through its specialized composition and structural architecture, while also modulating toughness via dynamic regulation of component ratios. Multiple TFs regulate SCW synthesis. A tertiary regulatory network governing SCW biosynthesis, composed of TFs from the NAC domain containing protein [66] and MYB domain protein (MYB) families, has been identified. This regulatory network exhibits a high degree of evolutionary conservation across species (Figure 4) [26,67,68]. Among the TFs involved in the primary level of network regulation, two major NAC subfamilies are predominantly represented: NAC Secondary Wall Thickening Promoting Factor or Secondary Wall-Associated NAC Domain Protein (SND), which includes *NST1*, *NST2*, *NST3*, and *SND2*, and the VND family members *VND1* through *VND7* (Figure 4). NAC TFs have been shown to play a predominant regulatory role in SCW biosynthesis in multiple plant species, including alfalfa and rice [69,70,71].

The secondary tier of the SCW biosynthetic transcriptional regulatory network is primarily regulated by *MYB46* and *MYB83*, which are directly activated by NST3 and other NAC family members (Figure 4) [72,73]. In *Arabidopsis*, *MYB46* and *MYB83* are expressed explicitly in fibers and vascular tissues undergoing SCW thickening, with a functionally redundant relationship. Double-mutant plants show reduced SCW thickness in the xylem, accompanied by severe growth retardation, wilting, and lethality (Figure 4) [72,73]. Like first-tier regulatory TFs, the functions of *MYB46* and *MYB83* are highly conserved across plants. In poplar, overexpression of either *PtrMYB3* or *PtrMYB20*, which are functionally homologous to *Arabidopsis MYB46* and *MYB83*, respectively, induces ectopic deposition of cellulose, xylan, and lignin in cortical and pith cells [73].

The third tier of the transcriptional regulatory network primarily comprises downstream TFs regulated by *MYB46* or *MYB83*, including *MYB43*, *MYB52*, *MYB54*, *MYB58*, *MYB63*, and *KNAT7* (Figure 4) [68]. Most third-tier TFs activate downstream SCW biosynthesis genes, promoting SCW biogenesis. However, specific TFs, such as *MYB7*, *MYB32*, *MYB4*, and *KNAT7*, function as negative regulators of SCW biosynthesis [74,75,76].

Collectively, this conserved, multilayered transcriptional network coordinates the precise spatial and temporal control of SCW formation in dicotyledonous plants. The integration of top-tier transcriptional regulators, intermediate amplifiers, and fine-tuning repressors enables flexible yet robust control of stem mechanical properties, providing a foundational framework for understanding SCW development across dicot species.

### 3.5. Genetic Regulation of Structural Components Underlying Stem Strength and Toughness

The synthesis of SCWs is a highly ordered process involving the coordinated activity of various enzymes and TFs. This process consists of three main stages: cellulose synthesis and deposition, hemicellulose synthesis and modification, and lignin polymerization and deposition. Cellulose synthesis constitutes the initial and most critical phase in SCW formation. Cellulose is synthesized by the Cellulose Synthase Complex (CSC), a multimeric enzyme complex composed of multiple CESA subunits. In *A. thaliana*, *CESA4*, *CESA7*, and *CESA8* assemble to form the CSC, which is essential for cellulose biosynthesis in SCWs (Figure 2) [77]. Mutations in these genes can lead to phenotypes such as increased stem brittleness, plant dwarfism, xylem tissue collapse, and aberrant growth patterns [78,79]. These observations highlight the indispensable role of SCW-associated CESAs in maintaining stem mechanical strength in dicot species.

Hemicelluloses are synthesized in the Golgi apparatus and transported to the SCW [26]. Multiple Golgi-localized glycosyltransferase families in *Arabidopsis* participate in the biosynthesis of the hemicellulose backbone and the addition of side chains. For example, irregular xylem *9* (*IRX9*) and *IRX14*, along with *IRX10* and *IRX7*, belong to the GT43 and GT47 glycosyltransferase families, respectively, and are responsible for elongating the xylan backbone. Mutations in these genes result in reduced xylan content and compromised secondary wall integrity within xylem tissues [80,81], underscoring the importance of hemicellulose composition in stem strength.

Lignin is deposited in the SCW via the oxidative polymerization of monolignols, a process catalyzed primarily by laccases (LACs) and peroxidases (PRXs) across diverse cell types [82]. In *Arabidopsis*, higher-order mutants such as *lac4 lac11 lac17* exhibit severe growth defects accompanied by markedly reduced lignin deposition, illustrating the essential role of LAC-mediated lignification in stem mechanical performance [83]. Similar roles for peroxidases have also been reported across diverse dicot tissues, further emphasizing the conserved nature of lignin polymerization mechanisms.

Collectively, these studies in dicotyledonous model systems demonstrate that genes involved in cellulose, hemicellulose, and lignin biosynthesis constitute core determinants of SCW integrity and stem mechanical properties. These conserved structural and enzymatic components provide a molecular framework for understanding how stem strength and toughness are established across dicot species.

## 4. Translational Implications for Soybean Stem Development

Although the regulatory networks governing stem strength and toughness are increasingly well defined in *Arabidopsis* and other dicotyledonous model species [84,85], their translation to soybean remains at an early stage. Comparative genomic analyses indicate that most regulators of vascular development and SCW biosynthesis are conserved in soybean, often as expanded multi-gene families resulting from polyploidy [86]. While key transcriptional cascades, such as NAC-MYB networks, and enzymes involved in cellulose and lignin biosynthesis show strong sequence conservation, their functional roles, regulatory interactions [87,88], and spatiotemporal expression patterns in soybean have not been systematically validated. Functional redundancy among paralogs further complicates direct extrapolation from model systems.

**Figure 5:**
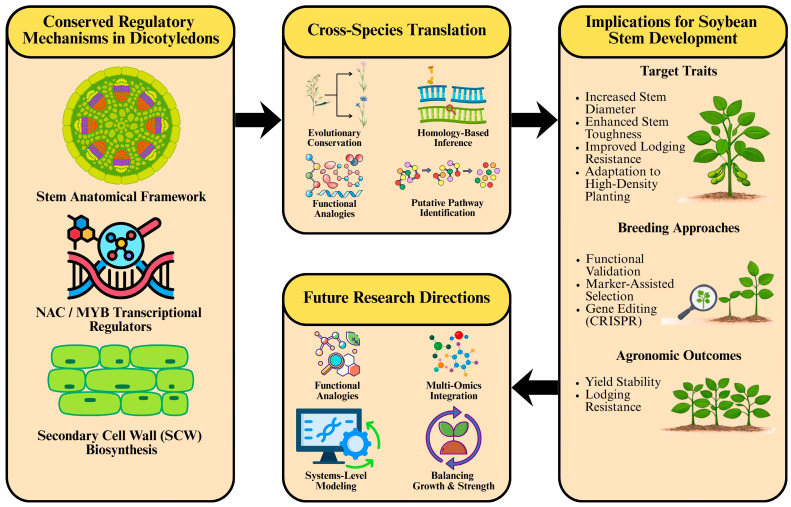
Translational framework linking conserved stem regulatory mechanisms in dicotyledonous plants to soybean improvement. Conserved stem regulatory modules identified in *Arabidopsis* and other dicots, including stem anatomical organization, NAC-MYB transcriptional regulation, and SCW biosynthesis pathways, provide a knowledge base for cross-species translation. Evolutionary conservation and homology-based inference enable the identification of putative regulatory modules relevant to soybean stem development. These insights support breeding and research strategies to enhance stem diameter, mechanical strength, and lodging resistance, ultimately improving agronomic performance. [Schematic diagram of the proposed mechanism (created with Canva 1.95.0, https://www.canva.cn)].

As illustrated in Figure 5, insights derived from conserved regulatory frameworks in dicotyledonous model species provide a conceptual basis for translating stem developmental mechanisms to soybean. However, the extent to which these conserved modules can be directly leveraged for trait improvement remains dependent on functional validation within the soybean genetic and developmental context.

Future translational efforts should therefore focus on systematically identifying and functionally characterizing conserved and soybean-specific regulatory genes involved in vascular differentiation, SCW formation, and cell wall modification. Genome editing technologies provide powerful tools to interrogate and fine-tune candidate regulators, either by disrupting negative regulatory elements or by modulating the expression of positive regulators [89,90]. Given that stem mechanical properties are developmentally dynamic, precise temporal and spatial control of gene activity will be essential to balance stem reinforcement with growth and developmental demands. Integrating functional genomics, genome editing, and predictive modeling will be critical for establishing effective strategies to improve soybean stem mechanical performance in future breeding programs.

### 4.1. Conserved and Candidate Regulatory Networks Relevant to Soybean Stem Mechanics

Compared with the model plant *A. thaliana*, relatively fewer genes associated with stem strength and toughness have been functionally characterized in soybean. Nevertheless, several regulators influencing stem mechanical properties and plant architecture have been identified. For example, editing of the gibberellin (GA) receptor gene *GmGID1* increases stem thickness and enhances soybean yield and nitrogen fixation capacity [91]. *GmRAV1* regulates stem regeneration through the CK signaling pathway [92], while *GmPIN1*-mediated auxin asymmetry controls petiole angle and overall plant architecture [93]. In addition, the *CS1*-encoded HEAT-repeat protein regulates amyloplast sedimentation, thereby modulating auxin polar transport and promoting xylem development as well as cellulose and lignin biosynthesis [94]. Despite these advances, many additional genes contributing to soybean stem strength and toughness remain to be identified.

Recent integrative genomic and transcriptomic studies have begun to uncover conserved and candidate regulatory networks that underpin stem mechanical properties in soybean. Liu et al. identified a major quantitative trait locus (QTL) spanning 15 genes, among which a WUSCHEL-related homeobox 4-like TF (GmWOX4-like) was highlighted as a high-confidence candidate for regulating stem strength based on integrated bulk transcriptome sequencing and single-cell RNA sequencing analyses [95].

Functional annotation of genes within this locus revealed coordinated, multi-level regulation of stem mechanical properties in soybean, involving transcriptional, post-transcriptional, and post-translational processes [95]. For example, *Long-Chain Base 1* (*GmLCB1*) regulates sphingolipid biosynthesis through multi-enzyme complexes and promotes the formation of higher-order oligomers that may influence cellular signaling pathways associated with stem development [96]. The identification of E3 ubiquitin ligases further suggests the involvement of post-translational regulation, consistent with findings that the soybean E3 ligase *Increased Leaf Petiole Angle 1* (*GmILPA1*) modulates plant architecture through degradation of the GA catabolic enzyme *GA2 OXIDASE-like* (*GmGA2ox-like*) [97]. In addition, the *Protein-Only RNA enzyme P1* (*GmPRORP1*), which plays a critical role in tRNA precursor processing, implicates post-transcriptional regulation in stem mechanical properties [98].

Spatial single-cell RNA sequencing demonstrated that *GmWOX4-like* is expressed in specific vascular cell types, particularly in the procambium and phloem. Integration of anatomical measurements with correlation analyses of xylem-to-phloem ratios suggested that *GmWOX4-like* may regulate phloem proportion, a key determinant of stem strength and morphology [95]. Co-expression network analyses further indicated that genes associated with cortical microtubule organization and cell wall biosynthesis may function downstream of *GmWOX4-like*. In addition, signaling pathways related to jasmonic acid (JA), potassium ion transport, and secondary metabolite biosynthesis may act upstream or downstream of this regulator, highlighting its integration into broader regulatory networks [99]. Natural variation analyses revealed that polymorphisms in the *GmWOX4-like* promoter region are associated with lodging rate variation, suggesting that specific haplotypes may serve as biomarkers of lodging resistance [95]. In addition, *GmCS1* has been reported to mediate stem morphology regulation in soybean [94].

Additional insight was provided by Liang et al. through whole-genome resequencing of 338 soybean accessions combined with genome-wide association studies (GWAS), which identified 13 stable loci associated with stem-related traits under two planting densities [100]. Among the candidate genes, *Glyma.19G215500*, a member of the GH3 family, encodes a β-xylosidase-like (BXL) protein involved in hemicellulose degradation [101]. Its *Arabidopsis* homolog *AtBXL2* is highly expressed in nodes and internodes, and studies in poplar have demonstrated roles in SCW formation and hemicellulose metabolism, suggesting a conserved function in regulating cell wall plasticity and lodging resistance [102].

Additional candidates include *Glyma.19G216600* (*SWN*), which encodes a Polycomb group protein forming regulatory complexes with VRN2, VIN3, and CLF and participates in SCW biosynthesis [103]. *Glyma.19G212700* (*GH9B13*), identified through integrated expression profiling, literature review, and haplotype analysis, encodes a glycosyl hydrolase homologous to *AtGH9B13*, which is involved in cellulose microfibril rearrangement during cellulose biosynthesis [100,104]. Although functional evidence remains limited, this gene has been proposed to influence cellulose crystallinity and synthesis in soybean stems. In addition, *Glyma.19G212800* (*SUS3*) encodes sucrose synthase, which catalyzes the reversible conversion of sucrose and UDP into UDP-glucose, a direct substrate for cellulose synthase. Evidence from cotton fiber development indicates that sucrose synthase supplies UDP-glucose to support cellulose biosynthesis [105], and *Glyma.19G212800* has therefore been implicated in soybean lodging regulation through its role in stem carbohydrate metabolism [100].

Collectively, these studies identify a suite of conserved and candidate regulatory genes associated with vascular development, cell wall biosynthesis, and stem mechanical strength in soybean, providing a genetic framework for future functional validation and translational breeding efforts.

### 4.2. Challenges in Direct Translation to Soybean

*A. thaliana* is an annual herbaceous plant belonging to the *Brassicaceae* family. It is characterized by a compact genome of approximately 125 Mb distributed across five chromosomes, with relatively low gene redundancy [106]. In contrast, soybean (*G. max*), an annual crop species derived from a perennial ancestor in the *Fabaceae* family, has experienced two rounds of whole-genome duplication (WGD) [107], resulting in a much larger genome of approximately 1.1 Gb comprising 20 chromosomes [108]. These duplication events have driven extensive gene family expansion, leading to a high prevalence of paralogous genes in the soybean genome. The realization of gene functions depends on their expression patterns (temporal-spatial specificity and expression intensity), which are determined by cis-regulatory elements such as promoters, enhancers, and insulators. These elements exhibit strong species specificity [109]. Due to differences in evolution and genetic backgrounds, gene homology does not necessarily imply functional consistency. This disparity provides clear implications for the cross-species application of *Arabidopsis* genes to soybeans. Relying solely on sequence homology to screen target genes is no longer sufficient to ensure functional effectiveness [110]. Clarify the functional differentiation characteristics of soybean homologous genes and integrate spatio-temporal transcriptomics [111]. Identify homologous genes associated with the strength and toughness of *A. thaliana* stems and specifically expressed in the stems. These genes may have higher functional similarity.

## 5. Improving Lodging Resistance and Plant Architecture in Soybean Breeding

Enhancing the strength and toughness of soybean stems increases their mechanical resistance to wind-induced forces, thereby reducing stem breakage and subsequent lodging under abiotic stresses such as wind and rain, ultimately minimizing yield losses [100,112,113]. Research indicates that the optimal soybean plant architecture is characterized by a plant height of 80 to 110 cm, an internode length of 3.5 to 4.5 cm, a total of 16 to 20 internodes, and relatively larger stem diameters [114,115]. Based on this, the population structure of soybean plants can be optimized to overcome the limitations of traditional dense planting, enable rational close planting, improve field ventilation and light penetration, and promote the efficient synthesis, transport, and distribution of photosynthetic products [7]. Moreover, it provides critical agronomic trait support for high-yield and stable-yield breeding programs and holds significant, irreplaceable value in advancing high-yielding soybean breeding.

Stem lodging resistance is a critical determinant of soybean yield stability, yet the genetic mechanisms underlying stem mechanical integrity and lodging tolerance remain incompletely understood. Sun et al. identified 12 QTLs associated with stem diameter across eight chromosomes using three recombinant inbred line (RIL) populations evaluated in five environments [116]. Among these loci, *q19* exhibited pleiotropic effects on both lodging resistance and yield [116].

Within the *q19* interval, a candidate gene encoding a squamosa promoter binding protein-like (SPL) TF (*Glyma.19G146000*) was proposed. Homologous SPL genes have been shown to reduce tiller number, enhance stem robustness, and improve lodging resistance in rice [117]. In addition, another candidate gene in this region, *Glyma.19G195400*, encodes soluble acid invertase-1 (SAI-1) and has been reported to regulate internode formation and elongation, thereby increasing stem thickness and lodging resistance in sorghum [116].

Complementing these quantitative genetic findings, Ye et al. characterized a lodging-related mutant, *lodging-related mutant 3* (*lrm3*), which exhibits reduced stem strength and increased lodging susceptibility [112]. *LRM3* knockout alleles were generated using CRISPR/Cas9-mediated gene editing, enabling functional validation of its role in stem mechanics. Molecular cloning revealed that *LRM3* encodes a U-box E3 ubiquitin ligase that interacts with the TF MYB6 and targets it for degradation via the 26S proteasome. Transcriptomic and chromatin immunoprecipitation analyses demonstrated that *MYB6* directly represses phenylalanine ammonia-lyase (PAL) gene expression, resulting in reduced lignin biosynthesis and diminished SCW deposition in soybean stems. Population genetic analyses further identified three major LRM3 haplotypes, with Haplotype 1 preferentially retained in landraces and modern cultivars, suggesting selection during soybean domestication [112].

Together, these studies highlight multiple genetic entry points, ranging from QTL-defined candidate genes to regulatory modules integrating ubiquitin-mediated proteolysis and phenylpropanoid metabolism, that contribute to stem mechanical strength, lodging resistance, and plant architecture in soybean. These findings provide valuable molecular targets and genetic resources for the development of lodging-resistant soybean cultivars through modern breeding strategies.

## 6. Conclusions and Future Perspectives

This review synthesizes current anatomical, genetic, and molecular evidence underlying stem strength and mechanical resilience in dicotyledonous plants, with a focus on their translational relevance for improving soybean lodging resistance. Comparative analyses indicate that key regulators of vascular development and SCW biosynthesis identified in model species are conserved mainly in soybean, although frequently expanded into multi-gene families due to polyploidy. This conservation provides a strong foundation for translational research but also necessitates systematic functional validation in soybean.

Emerging evidence highlights several priority regulatory modules for improving soybean stem mechanics. Central among these are conserved NAC-MYB transcriptional cascades, which coordinate SCW deposition by regulating downstream biosynthetic pathways. Functional studies demonstrate that lignin biosynthesis enzymes, particularly PAL, act as critical determinants of stem rigidity, as exemplified by the LRM3-MYB6 regulatory module, in which ubiquitin-mediated degradation of *MYB6* modulates *PAL* expression and lignin accumulation. In parallel, regulators influencing hormone signaling and carbon allocation, including *GmGID1* (gibberellin signaling), *CS1* (auxin-mediated vascular development), and *SAI-1* (internode elongation and stem thickness), further underscore the multifactorial control of stem mechanical properties.

Quantitative genetic studies have identified multiple QTLs associated with stem diameter, lodging resistance, and yield stability, with loci such as *q19* revealing pleiotropic effects on both structural resilience and productivity. Candidate genes within these regions, including *GmWOX4-like*, *SPL* TFs, and post-transcriptional regulators such as *GmPRORP1*, illustrate the diverse regulatory layers, transcriptional, hormonal, metabolic, and post-transcriptional, that collectively shape stem strength and architecture.

Looking forward, translational efforts should prioritize integrating comparative genomics, single-cell transcriptomics, and genome editing to resolve functional redundancy among paralogs and fine-tune SCW-related pathways. CRISPR/Cas-based multi-gene editing, combined with marker-assisted selection, offers a powerful strategy to balance stem strength and flexibility without compromising growth or yield. Collectively, targeted manipulation of conserved transcriptional networks and SCW biosynthetic enzymes provides a rational path toward developing lodging-resistant soybean cultivars with improved yield stability under high-density planting and increasingly variable environmental conditions.

## Data Availability

No new data were created or analyzed in this study. Data sharing is not applicable to this article.
